# Motor Stereotypies: A Pathophysiological Review

**DOI:** 10.3389/fnins.2017.00171

**Published:** 2017-03-29

**Authors:** Zsanett Péter, Melody E. Oliphant, Thomas V. Fernandez

**Affiliations:** ^1^Department of Biology, Sewanee: The University of the SouthSewanee, TN, USA; ^2^Department of Chemistry, Sewanee: The University of the SouthSewanee, TN, USA; ^3^Yale Child Study Center, Yale University School of MedicineNew Haven, CT, USA; ^4^Department of Psychiatry, Yale Child Study Center, Yale University School of MedicineNew Haven, CT, USA

**Keywords:** motor stereotypies, dopamine, GABA, acetylcholine, environment, genetics, pharmacotherapy, brain imaging

## Abstract

Motor stereotypies are common, repetitive, rhythmic movements with typical onset in early childhood. While most often described in children with autism spectrum disorder (ASD) and intellectual disability (ID), stereotypies can also present without developmental delay and persist into adulthood. Stereotypies are often disruptive and harmful, both physically and socially, and effective evidence-based treatments are lacking. This can be attributed, in part, to our incomplete knowledge of the underlying biological and environmental risk. Several studies implicate various neurotransmitters, brain circuits, anatomical loci, and pre- and post-natal environmental influences in stereotypy onset and symptom severity. However, there are few points of convergence among a relatively small number of studies, indicating that more research is needed to confirm the underlying bases of risk. Of particular note is the lack of published genetic studies of stereotypies, despite evidence for Mendelian inheritance patterns in some families. Focusing future studies on typically-developing children with primary motor stereotypies may be a useful approach to minimize potential biological, environmental, and genetic heterogeneity that could theoretically hinder consistent findings. Ultimately, a deeper understanding of the underlying biology and risk factors for motor stereotypies will lead us closer to more effective targeted therapies that will alleviate suffering in affected children.

## Introduction

Motor stereotypies are repetitive, rhythmic, often bilateral movements with a fixed pattern (e.g., hand flapping, waving, or rotating) and regular frequency that can usually be stopped by distraction (e.g., calling one's name) (Harris et al., 2008). In the scientific literature, stereotypies often refer not only to movements, but also to other behaviors (e.g., postures, utterances, sniffing) that are classified as repetitive (Symons et al., 2005; Carcani-Rathwell et al., 2006). Stereotypies are most often recognized in children with autism spectrum disorder (ASD), sensory deprivation, or intellectual disability (ID), but they are also observed in typically-developing children (Singer, 2009). Stereotypies can be classified as primary, meaning that they appear to be purely physiological, or secondary, existing in association with other psychiatric or neurological disorders (Muthugovindan and Singer, 2009). This review will focus on summarizing what is known about biological and environmental risk factors associated with stereotypies in order to gain a deeper understanding about their possible underlying causes and to identify knowledge gaps that should be addressed in future studies.

Primary stereotypies can be classified into two groups, common (e.g., pencil tapping, hair twisting, nail biting) and complex (e.g., hand flapping, waving, finger wiggling etc.) (Singer, 2009). About 20% of children exhibit common types of primary motor stereotypies, while primary complex motor stereotypies are estimated to affect 3–4% of children in the U.S. (Singer, 2009). The typical age of onset for motor stereotypies is before 3 years, with 80% of cases exhibiting repetitive movements by age 2 (Harris et al., 2008; Singer, 2009). Motor stereotypies usually occur when a child is engrossed in an activity or experiencing excitement, stress, boredom, or fatigue. They may last for seconds to minutes and are completely absent during sleep (Singer, 2009). Children may report feeling satisfied and happy when exhibiting stereotypies, while others may be unaware of their own stereotypies and do not consider them to be disruptive or anxiety-provoking (Singer, 2013). However, in many cases, motor stereotypies can be self-injurious, socially offensive, or disruptive to desired activities (Maraganore et al., 1991; Symons et al., 2005).

Secondary stereotypies most often occur in children with ASD. Forty-four percent of patients with ASD report some form of stereotypic movement. Furthermore, the severity and frequency of motor stereotypies in ASD is correlated with severity of illness, degree of ID, and impairments in adaptive functioning and symbolic play. The frequency of self-injurious motor stereotypies is higher in individuals diagnosed with ASD compared to children with typical development, and highest among children who are diagnosed with both ASD and ID (Ghanizadeh, 2010).

Unlike tic disorders, children usually report no premonitory urges prior to their motor stereotypies and therefore cannot use this sensation to trigger efforts to suppress their movements (Singer, 2009). Stereotypies usually persist into adulthood, and only a small percentage of affected individuals are able to control them. A longitudinal study that followed 100 children for up to 10 years found that 94% of the participants continued to experience stereotypic movements. Only 3% of children with hand or arm movements managed to suppress stereotypies completely, while nearly one-third of children with head movements (3 out of 8 in the longitudinal study) were able to leave these repetitive movements behind. Over 50% of patients reported no change in the severity of stereotypies over the monitored years, while 10% reported worsening of the symptoms (Harris et al., 2008).

Comorbid attention-deficit/hyperactivity disorder (ADHD), tics, and obsessive-compulsive disorder (OCD) are reported to occur frequently in patients with complex motor stereotypies (Harris et al., 2008). In one study, 50% of typically developing children experienced some disorder in combination with their stereotypies: ADHD (30%), tics (18%), OCD and obsessive-compulsive behaviors (10%) and Tourette's disorder (7%) (Harris et al., 2008).

Although the pathophysiology of motor stereotypies is not fully understood, both biological (genetic, brain structure and function, neurochemical) and psychological factors have been investigated in the etiology of these movements. Psychological hypotheses emphasize compensation for external sensory deficits, channeling of thoughts from excess capabilities into movements, substituting for imaginary activities, or executing movements as part of obsessive-compulsive or anxiety-related behaviors (Maraganore et al., 1991; Singer, 2009). While psychological factors may play some contributory role (Singer, 2009), there exists far more evidence supporting a biological basis, so we will focus the remainder of this review on evidence for a biological diathesis.

## Neurochemical abnormalities

Most neurobiological clues in motor stereotypies have been discovered in animal models, where stereotypies can be induced by drugs that stimulate dopamine release (e.g., amphetamines), dopamine reuptake inhibitors (e.g., cocaine), as well as direct dopamine receptor agonists (e.g., apomorphine) (Canales and Graybiel, 2000; Aliane et al., 2011). Converging with these findings is the observation that elderly adults with ID who engage in stereotypic movements exhibit lower rates of eye-blinking and greater variability in eye blinking intervals. Given that eye-blinking rate has been found to directly correlate with dopamine function, the above findings further suggest that stereotypies are linked to dopaminergic dysfunction (Roebel and MacLean, 2007).

However, dopamine is not the only neurotransmitter believed to contribute to the onset of stereotypies. γ-Aminobutyric acid (GABA) involvement in complex motor stereotypies was discovered through a recent proton magnetic resonance imaging study (Harris et al., 2016). Lower levels of GABA were observed in the anterior cingulate cortex (ACC), a brain region responsible for emotional regulation, and in the striatum, involved in motor and action planning, decision-making, motivation, reinforcement, and reward perception. Furthermore, levels of GABA in the cingulate cortex were a predictor of symptom severity (Harris et al., 2016).

Within the corticostriatal brain circuit of animals with drug-induced stereotypies, increased levels of extracellular dopamine in the dorsal striatum was associated with decreased levels of acetylcholine (ACh) release in this region; this imbalance of dopamine and ACh correlated with severity of motor stereotypies. In Sprague-Dawley rat models, restoring optimal cholinergic transmission with acetylcholinesterase inhibitors in the prefrontal striatal region led to motor stereotypy arrest (Aliane et al., 2011). When degeneration of cholinergic neurons was induced or when the post-synaptic effects of ACh were blocked, motor stereotypies were increased. Furthermore, raclopride, a dopamine D2 antagonist, caused arrest of stereotypic movements when injected in the prefrontal dorsal striatum, with no effect when injected in the sensorimotor area of the dorsal striatum (Aliane et al., 2011). These findings suggest one distinct neurological basis for stereotypies, and this knowledge provides rationale for an ongoing clinical trial studying the use of acetylcholinesterase inhibitors in children with ASD (ClinicalTrials.gov ID NCT01098383). Increased levels of glutamate and aspartate in the striatum have also been observed during stereotypies in mouse models (Presti et al., 2004).

## Neural circuit function and structural abnormalities

The cortical-striatal-thalamo-cortical (CSTC) brain circuit is thought to be involved in the manifestation of motor stereotypies. These processing loops have long been linked to movement initiation, continuation, termination and other psychiatric disorders such as OCD, ADHD, and Tourette's disorder, all of which are frequently comorbid with complex motor stereotypies (Gao and Singer, 2013). Significant activational imbalance in the prefrontal territory of the dorsal striatum has been found in rats with induced motor stereotypies (Aliane et al., 2011). Specifically, the ratio of increased activation in the striosome within the striatum, the main provider of input to the basal ganglia, to the extrastriosomal matrix, called the striomal predominance value, is a strong predictive measure of the severity of stereotypies (Canales and Graybiel, 2000). Supportive of this finding is a case study of a 17-year old male who suffered a right putamen infarction, followed by the sudden onset of motor stereotypies. Together, these findings suggest a basal ganglia dysfunction in individuals with motor stereotypies (Maraganore et al., 1991; Canales and Graybiel, 2000; Singer, 2009; Langen et al., 2011).

A functional MRI study involving subjects with ASD found a direct correlation between functional changes in the ACC and repetitive behaviors. More specifically, the study found deficient response monitoring in children with ASD, as these subjects made more errors on antisaccade tasks and had a more difficult time differentiating between erroneous and right answers, as well as higher activation in the ACC (Thakkar et al., 2008).

Based on altered function, several brain regions are believed to underlie motor stereotypies. Similarly, studies of brain structural variation in subjects with motor stereotypies have suggested several anatomical loci (Gao and Singer, 2013). One structural MRI study found decreased striatal volume (Goldman et al., 2013), while another found reduced bilateral caudate nucleus volumes (Kates et al., 2005). Ambiguity exists about potential volume changes in the basal ganglia, as some studies find a larger volume, while others observe no change or decreased volume (Langen et al., 2011; Goldman et al., 2013). Larger caudate nucleus and putamen volumes were shown to correlate with repetitiveness, and cerebellar white matter volume was found to correlate with severity of symptoms (Ghanizadeh, 2010). A volumetric MRI study of 9 males with complex motor stereotypies also suggested an inverse correlation between symptom severity and volume of frontal and temporal white matter when adjusted for cerebral size (Kates et al., 2005). Finally, a study in ASD subjects, which found neural activation differences in the ACC, also reported structural differences in this region (Thakkar et al., 2008).

## Genetic risk

A study of 100 typically-developing children with motor stereotypies found that 25% of first- or second-degree relatives had a positive family history that was limited to either the maternal or paternal lineage with relatively equal distribution, while 75% of cases appeared to be sporadic (Harris et al., 2008). In light of these findings, it is hypothesized that detection of both inherited and spontaneous (*de novo*) mutations could be highly productive approaches to gene discovery, similar to the utility of these approaches in prior studies of ASD (Sanders et al., 2015; Leppa et al., 2016) and schizophrenia (Fromer et al., 2014; McCarthy et al., 2014). To date, no gene variants have been reported that increase the risk for stereotypies, yet these studies are underway in our lab and others. One path toward gene discovery is studying families with primary stereotypies in otherwise typically developing children who lack major developmental comorbidities. This may be a more efficient path to gene discovery, since typically developing children with stereotypies may represent a genetically more homogenous group of individuals versus those with secondary stereotypies with developmental delay. These genetic studies have potential to reveal specific genes, biological pathways, and critical developmental time periods contributing to motor stereotypies in children both without and with social disability, which would greatly advance our knowledge of their underlying biology and potential treatment targets.

## Environmental risk

Environmental factors have also been found to affect the development and severity of stereotypies. Most of the studies that investigated environmental influences focused on primate animals. Non-human primates have the closest evolutionary path to humans and have a similarly prolonged developmental period and complex cognitive processing, social interactions, and behaviors (Lutz, 2014). Rearing conditions have been repeatedly implicated in the development of motor stereotypies (Lutz, 2014). Chimpanzees and rhesus macaques reared in isolation, and even those reared by peers instead of a mother, exhibit more stereotypies than those animals reared in a community by the mother (Davenport and Menzel, 1963; Mitchell, 1968). This phenomenon was also evident among humans in a study of Romanian orphanages, where a large portion of children (>80%) exhibited stereotypies, mostly in the form of body rocking, while those placed in foster care showed significantly reduced stereotypic behaviors (Fisher et al., 1997; Beckett et al., 2002; Hoksbergen et al., 2005; Bos et al., 2010).

Studies of environments lacking usual amounts of sensory input have also been shown to correlate with the severity of stereotypies. In both animal models and humans, stereotypies increased when subjects were placed in restricted environments (e.g., empty room, psychiatric institutions, orphanages) or separated from community versus placement in a natural environment with peers (Hutt and Hutt, 1965; Warren and Burns, 1970; Lutz, 2014). These movements and behaviors are often interpreted as self-stimulating, a commonly mentioned phenomenon in children with ASD, positing their function as maintaining an optimal level of stimulation (Lutz, 2014).

On the other hand, overstimulating environments may also induce stereotypies. In these cases, stereotypies may maintain homeostasis and reduce anxiety stemming from overstimulation (Lutz, 2014). The intense world hypothesis posits that some children with ASD experience overstimulation and exhibit repetitive behaviors to reduce high arousal from incoming sensory stimuli (Lutz, 2014). A study in ASD supports this hypothesis, as children exhibited increased severity and frequency of stereotypies when environmental cues were added to their surroundings (e.g., wooden block, passive adult observer, noise, etc.) (Hutt and Hutt, 1965).

In addition to postnatal environmental factors, prenatal circumstances might predispose toward a stereotypic phenotype as well. One hypothesized factor is prenatal stress. Although no clear evidence exists that offspring with stereotypic movements have higher levels of glucocorticoid hormones, indicating higher stress, it is believed that in utero chronic stress may possibly lead to stereotypies through some type of epigenetic mechanism (Mostard, 2011).

## Treatments

Currently, there are no established pharmacological treatments available for motor stereotypies. A wide range of medications have been utilized for treatment of motor stereotypies and associated self-injurious behaviors in ASD, but their efficacy is generally inconsistent and inadequate, and their use is limited by the potential for long-term adverse side effects. Several antipsychotics have been employed in an attempt to treat motor stereotypies in ASD (haloperidol, pimozide, clozapine, risperidone, olanzapine, ziprasidone, quetiapine, aripiprazole, and paliperidone), with haloperidol, risperidone and olanzapine shown as at least minimally efficacious in reducing stereotypic movements and behaviors, but with sometimes serious, impairing side effects (Ching and Pringsheim, 2012; Doyle and McDougle, 2012). Selective serotonin-reuptake inhibitors (SSRIs) and serotonin and norepinephrine reuptake inhibitors (SNRIs) (fluoxetine, clomipramine, fluvoxamine, sertraline, citalopram, escitalopram, venlafaxine, trazodone, and mirtazapine) have also been studied to treat ASD stereotypies. Clomipramine, fluvoxamine, fluoxetine, sertraline, citalopram and venlafaxine were found to be somewhat effective but the use of most of these medications were limited by unwanted side effects and the lack of adequately-powered studies to determine their effect (Doyle and McDougle, 2012). In practice, many psychotropic medications are prescribed for patients with motor stereotypies, but to date there is no established drug-based treatment. One longitudinal study followed 100 normally developing children with complex motor stereotypies for a mean duration of 6.8 ± 4.6 years. None of these children or their caregivers reported reduced frequency, duration, or amplitude of the stereotypic movements despite treatment with prescribed medications that included clonidine, risperidone, oxcarbazepine, fluoxetine, topiramate, pimozide, levetiracetam, divalproex, carbamazapine, clonazepam, phenytoin, and acetazolamide (Harris et al., 2008).

Cognitive behavioral therapy (CBT), specifically focusing on habit reversal and differential reinforcement, has some evidence of efficacy for primary complex motor stereotypies (Miller et al., 2006; Specht et al., 2017). Since CBT requires active participation of the child, they must demonstrate the cognitive capability to understand and follow advice from a therapist. This prerequisite will often exclude children with ID, who often experience the most severe and frequent symptoms, and those who are too young (Miller et al., 2006). Recently, a parent-guided DVD intervention was developed at Johns Hopkins University that focuses on suppressing complex motor stereotypies through behavioral therapy methods. In this study, children were encouraged to exhibit stereotypic movements intentionally to raise awareness and conscientiousness while performing these behaviors, and parents are trained to verbally reward their children when stereotypic behaviors did not occur. This intervention included 54 participants and was found to be beneficial for patients with stereotypies, significantly reducing scores on all motor stereotypy screening scales [Stereotypy Severity Scale (SSS) – Motor and Impairment Scores, Stereotypy Linear Analog Scales (SLAS)] (Specht et al., 2017).

In summary, medications are generally regarded as an ineffective treatment for primary motor stereotypies, so they are rarely prescribed. However, this practice is based mainly on anecdotal evidence (Tan et al., 1997; Oakley et al., 2015), as there have been no formal studies of medications for stereotypies in normally developing children to date. However, there is empirical evidence to support the benefits of both therapist- (Miller et al., 2006) and home-based (Specht et al., 2017) behavioral therapy programs for primary complex motor stereotypies, so this is the favored choice of therapy. Similarly, studies of pharmacotherapies for stereotypies in children with developmental delay have been inconsistent, as reviewed above, so there is no consensus on the best treatment approach for these children, who are often not candidates for behavioral therapies. There is an urgent need for further research into motor stereotypies across all modalities to guide us toward a better understanding of disease mechanisms, biomarkers, and treatment targets.

## Conclusion

Motor stereotypies occur in early childhood and are potentially disabling. They can present in otherwise typically-developing children, although they have been most often studied in children with ASD and ID. Limited improvement has been reported with existing pharmacological therapies, and more effective treatments are urgently needed. A prerequisite for the discovery of improved therapies is understanding the underlying pathophysiology, including biological mechanisms as well as biological and environmental risk factors. There has been some progress in this regard, but more studies are needed. In particular, there have been no published genetic studies of stereotypies, despite evidence for Mendelian inheritance patterns in some families. Genetic studies exhibit proven potential to signal risk genes, biological pathways and networks, and critical developmental time periods that can teach us about the underlying biology of complex neuropsychiatric phenotypes. Furthermore, progress may be facilitated by prioritizing future studies on typically-developing children with primary motor stereotypies, as these cases may represent more biological, environmental, and genetic homogeneity compared to children with secondary stereotypies. By building on the knowledge from past studies and using modern research methods, there is great potential to achieve a deeper understanding of stereotypies that will lead us toward prevention and effective treatments.

## Author contributions

ZP, MO, and TF contributed to the conceptualization, literature review, writing, and revision of this article.

## Funding

Ms. Péter receives tuition and stipend support from the Klein Family Scholarship and Sewanee International Scholarship. Ms. Oliphant has no financial disclosures. Dr. Fernandez has received research funding from NIMH, the Simons Foundation, Allison Family Foundation, and Shire.

### Conflict of interest statement

The authors declare that the research was conducted in the absence of any commercial or financial relationships that could be construed as a potential conflict of interest.
